# 1547. Integrating Long-acting Injectable Cabotegravir for PrEP into Standard of Care for Cisgender Women, Transgender Women, Transgender Men, and Men Who Have Sex with Men: Results from the PILLAR & EBONI Studies

**DOI:** 10.1093/ofid/ofad500.1382

**Published:** 2023-11-27

**Authors:** William M Valenti, John Phoenix, Rupa R Patel, Zandraetta Tims-Cook, Nanlesta Pilgrim, Amber Haley, Kenneth Sutton, Alison Gaudion, Deanna Merrill, Rimgaile Urbaityte, Bo Li, David Andrae, Courtney Bonner, Abigail Herbst, Dhuly Chowdhury, Harmony Garges, Maggie Czarnogorski

**Affiliations:** Trillium Health, Rochester, New York; Huntridge Family Clinic, Las Vegas, Nevada; Washington University in St. Louis; Faebris Medical & Community Education, Atlanta, Georgia; ViiV Healthcare, Durham, North Carolina; ViiV Healthcare, Durham, North Carolina; ViiV Healthcare, Durham, North Carolina; ViiV Healthcare, Durham, North Carolina; ViiV Healthcare, Durham, North Carolina; GSK, Uxbridge, England, United Kingdom; GSK, Uxbridge, England, United Kingdom; Evidera Inc., Bethesda, Maryland; RTI International, Research Triangle Park, North Carolina; Evidera Inc., Bethesda, Maryland; RTI International, Research Triangle Park, North Carolina; ViiV Healthcare, Durham, North Carolina; ViiV Healthcare, Durham, North Carolina

## Abstract

**Background:**

EBONI and PILLAR are Phase IV implementation science trials evaluating the integration of long-acting injectable Cabotegravir for PrEP (CAB LA) into standard of care at 37 clinics across the U.S. EBONI enrolls Black cisgender/transgender women and PILLAR enrolls men who have sex with men (MSM)/transgender men. We report CAB LA implementation plans among staff study participants (SSPs).

**Methods:**

Of 105 SSPs from 20 EBONI sites, 65 (62%) from 14 activated clinics completed baseline surveys starting in August 2022. Eighty-six (100%) SSPs from all 17 PILLAR clinic sites completed surveys from April-October 2022. Various clinic types were selected to enroll diverse populations. Survey domains included SSPs’ perceptions of implementing CAB LA, plans for integration, and estimation of resources needed for integration were collected.

**Results:**

Table 1 presents notable gender and racial differences among SSPs across the studies. Table 2 reveals similar SSPs’ experience with PrEP and injections within the two studies. A high proportion of SSPs (EBONI: 97%; PILLAR: 85%) felt extremely positive or positive about implementing CAB LA. The majority of SSPs in EBONI (74%) than in PILLAR (53%) perceived implementation would be very easy or easy.

Regarding CAB LA integration plans, compared to EBONI, SSPs in PILLAR reported being able to manage more CAB LA patients per week; having more staff prepared to give injections; having a specific person to follow up with no-shows; planning to have providers track patients; and planning to offer drop-in injection options (Table 3). However, more SSPs in EBONI reported plans to create roles to coordinate the CAB LA program.

Various appointment reminder tools are in place and used in similarly high proportions across both studies. However, a higher proportion of SSPs in PILLAR reported using email and postal reminders.

**Figure:**
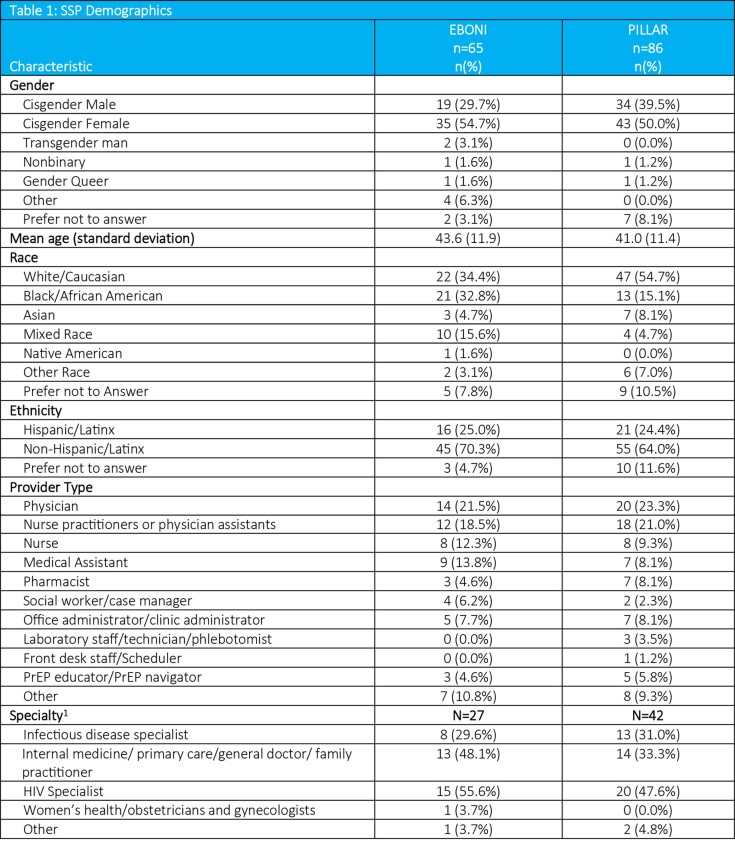

**Figure:**
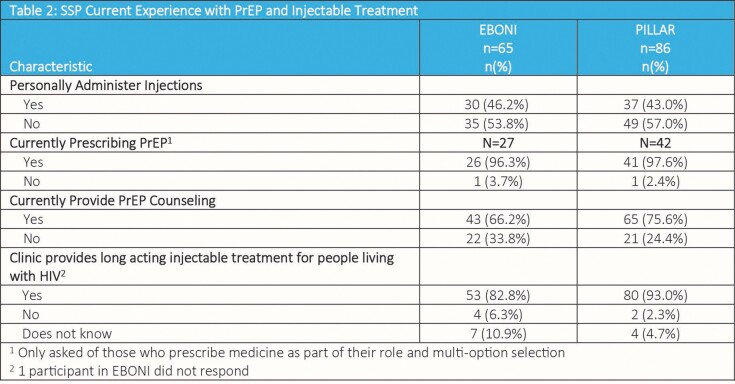

**Figure:**
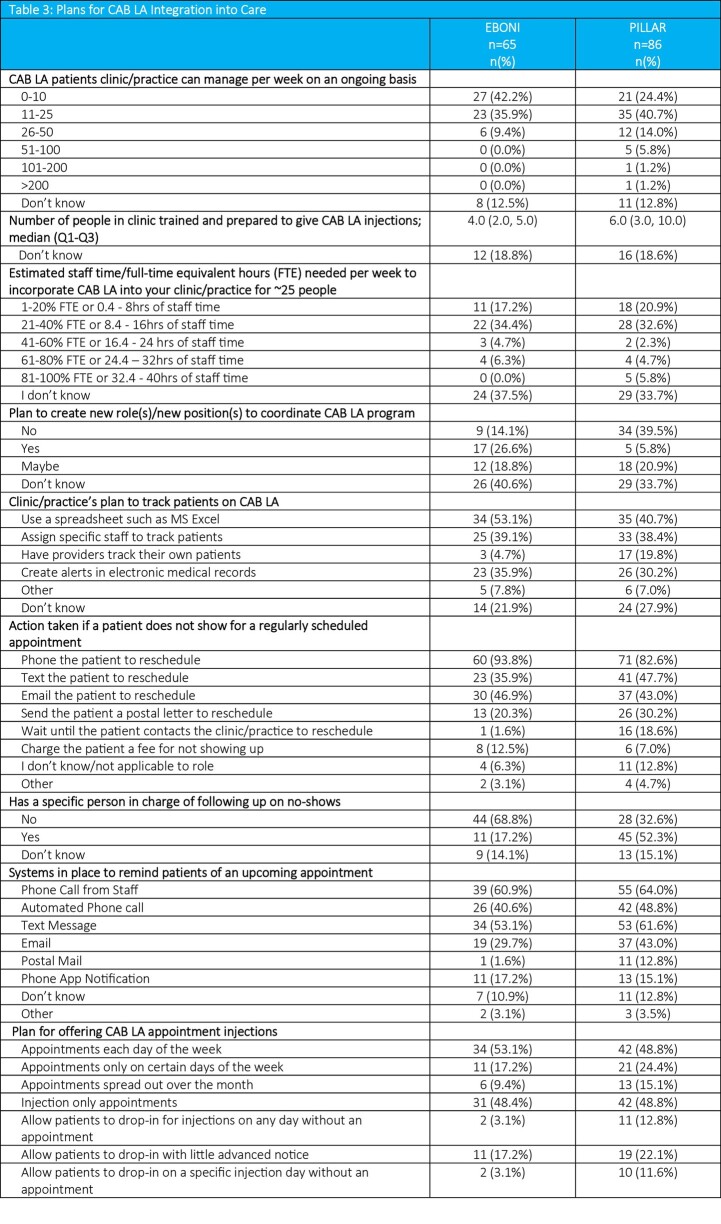

**Conclusion:**

Healthcare staff were positive about integrating CAB LA into care. Sites serving MSM and transgender men had more detailed implementation plans in place than those serving cisgender and transgender women. Sites serving women may need additional support to develop their plans to build staff and clinic capacity. To address the gaps identified in clinic planning, an equity-centered implementation strategy should be employed.

**Disclosures:**

**William M. Valenti, MD, FIDSA**, Gilead: Grant/Research Support|ViiV Healthcare: Grant/Research Support **John Phoenix, MSN, APRN, FNP-C**, Gilead: Grant/Research Support|Gilead: Speaker Bureau|Huntridge Family Clinic: Ownership Interest|Napo Pharmaceuticals: Speaker Bureau|ViiV Healthcare: Grant/Research Support|ViiV Healthcare: Speaker Bureau **Rupa R. Patel, MD MPH**, Gilead: Advisor/Consultant|Gilead: Grant/Research Support|Roche: Advisor/Consultant|ViiV Healthcare: Advisor/Consultant|ViiV Healthcare: Grant/Research Support **Zandraetta Tims-Cook, MD**, ViiV Healthcare: Speaker **Nanlesta Pilgrim, PhD**, ViiV Healthcare: Employment|ViiV Healthcare: Stocks/Bonds **Amber Haley, PhD**, ViiV Healthcare: Former Employment **Kenneth Sutton, MA**, ViiV Healthcare: Employment|ViiV Healthcare: Stocks/Bonds **Alison Gaudion, PhD**, ViiV Healthcare: Employment|ViiV Healthcare: Stocks/Bonds **Deanna Merrill, PharmD, MBA, AAHIVP**, ViiV Healthcare: Employment|ViiV Healthcare: Stocks/Bonds **Rimgaile Urbaityte, MSc**, GSK: Employment|GSK: Stocks/Bonds **Bo Li, PhD**, GSK: Employment|GSK: Stocks/Bonds **David Andrae, PhD**, Evidera: Employment **Abigail Herbst, MPH**, Evidera: Empoyment|ViiV Healthcare: Grant/Research Support **Harmony Garges, MD**, GSK: Stocks/Bonds|Medexus Pharmacy: Board Member|ViiV Healthcare: Empoyment **Maggie Czarnogorski, MD MPH**, ViiV Healthcare: Employment|ViiV Healthcare: Stocks/Bonds

